# Mitochondrial pyruvate carrier 1 mediates abscisic acid-regulated stomatal closure and the drought response by affecting cellular pyruvate content in *Arabidopsis thaliana*

**DOI:** 10.1186/s12870-017-1175-3

**Published:** 2017-11-22

**Authors:** Jian-Lin Shen, Chun-Long Li, Mei Wang, Li-Long He, Min-Yan Lin, Dong-Hua Chen, Wei Zhang

**Affiliations:** 1 Key Laboratory of Plant Cell Engineering and Germplasm Innovation, Ministry of Education, School of Life Science, Shandong University, Jinan, 250100 China; 2 College of Life Science, Jiangsu Normal University, Xuzhou, 221116 China

**Keywords:** Aba, *MPC1*, Guard cells, Stomatal closure, Anion currents, ROS, Drought resistance

## Abstract

**Background:**

Stomata are micropores surrounded by pairs of guard cells, and their opening is finely controlled to balance water vapor loss as transpiration and CO_2_ absorption for photosynthesis. The regulatory signaling network for stomatal movement is complicated, and increasing numbers of new genes have been shown to be involved in this process. Our previous study indicated that a member of the plant putative mitochondrial pyruvate carrier (MPC) family, NRGA1, is a negative regulator of guard cell abscisic acid (ABA) signaling. In this study, we identified novel physiological roles of pyruvate and MPC1, another member of the MPC family, in the regulation of stomatal closure in *Arabidopsis*.

**Results:**

Loss-of-function mutants of *MPC1* (*mpc1*) were hypersensitive to ABA-induced stomatal closure and ABA-activated guard cell slow-type anion currents, and showed a reduced rate of water loss upon drought treatment compared with wild-type plants. In contrast, plants overexpressing *MPC1* showed a hyposensitive ABA response and increased sensitivity to drought stress. In addition, *mpc1* mutants accumulated more pyruvate after drought or ABA treatment. The increased pyruvate content also induced stomatal closure and activated the slow-type anion channels of guard cells, and this process was dependent on the function of RbohD/F NADPH oxidases and reactive oxygen species concentrations in guard cells.

**Conclusions:**

Our findings revealed the essential roles of MPC1 and pyruvate in stomatal movement and plant drought resistance.

**Electronic supplementary material:**

The online version of this article (10.1186/s12870-017-1175-3) contains supplementary material, which is available to authorized users.

## Background

Drought is probably the most frequently encountered abiotic stress that limits plant development and growth [1]. Among the diverse pathways that plants have evolved to either tolerate or adapt to this stress, the control of stomatal aperture by regulating turgor in the pair of guard cells surrounding each stoma is perhaps the most important [2, 3]. Although the stomata are anatomically simple, the surrounding guard cells are morphologically distinct from epidermal cells and exhibit complex signal transduction networks that influence stomatal aperture. These features allow rapid modulation of guard cell turgor in response to a diverse set of biotic or abiotic stimuli, including light, CO_2_, pathogen infection, and plant hormones, promoting stomatal movement at time scales of seconds to hours [4, 5].

Stomatal movement is responsive to the local concentration of the phytohormone abscisic acid (ABA) [6–8]. The ABA-triggered activation of guard cell anion channels results in the efflux of anions, which in turn reduces the turgor of the guard cells to close the stomata [9–11]. In addition, the cytosol reactive oxygen species (ROS) is elevated in response to ABA accumulation, and is involved in ABA-regulated transmembrane ion trafficking of guard cells to close stomata [12–15], and the NADPH oxidases, e.g. RbohD and RbohF, are needed in the ABA activated ROS production [16]. With regard to the complex signaling networks influencing stomatal aperture, many novel functional genes, proteins, and factors were found to be involved in the molecular mechanisms of ABA signaling and stomatal movement [17].

Recently, we identified Negative Regulator of Guard cell ABA signaling 1 (NRGA1) as a putative mitochondrial pyruvate carrier (MPC) that negatively regulates ABA-induced guard cell signaling in *Arabidopsis* [18]. MPC proteins, which were first identified by two independent research groups using genetic and bioinformatics approaches in 2012 [19, 20], are highly conserved from yeast to humans and are necessary for the uptake of pyruvate in the inner mitochondrial membrane. Pyruvate, as the end product of glycolysis, is derived from sources in the cellular cytoplasm, and the majority is transported into mitochondria for oxidative metabolism through the tricarboxylic acid (TCA) cycle [21]. As the gatekeeper for pyruvate transport, mitochondrial pyruvate uptake was shown to be regulated by alteration of pyruvate carrier complexes between different MPC subunits in yeast [22]. Alba also reported that the regulation of mitochondrial pyruvate uptake is an important determinant of respiration rate and stress resistance in yeast [23]. In the field of human health, recent molecularly targeted research of the MPC confirmed its importance in both gluconeogenesis in diabetes [24] and proliferation of cancer cells [25, 26].

In contrast to the known functions of MPC in humans and yeast, only one MPC2-like protein, NRGA1, has been shown to be involved in stomata ABA signaling in *Arabidopsis* [18]. In addition to NRGA1, the *A. thaliana* genome encodes four other MPC candidates, but little is known regarding their functions [27]. Here, the *Arabidopsis* MPC1 (Gene ID: 832,131) was shown to interact with NRGA1 and to play roles in regulation of stomatal movement and pyruvate content. Moreover, these findings suggested that pyruvate could also be a cellular signal involved in anion channel activation and thus promotion of stomatal closure in a ROS-dependent manner, and they provided a possible mechanism by which AtMPC1 controls stomatal movement and the drought response.

## Methods

### Plant materials and growth conditions

Columbia-0 (Col-0) *A. thaliana* was used as the wild-type strain in this study; Col-0 is also the background of the T-DNA insertion mutant *mpc1* (SALK_008945, obtained from ABRC, http://www.abrc.osu.edu). The double mutant *mpc1/nrga1* was obtained by hybridization between *mpc1* and *nrga1* (SALK_050950, obtained from ABRC, http://www.abrc.osu.edu). Seeds were surface-sterilized with 75% ethanol for 3 min, followed by 95% ethanol for 1 min, and finally air-dried before use. The surface-sterilized seeds were subsequently plated on agar-solidified half-strength Murashige and Skoog (1962) medium (1/2 MS), maintained for 3 days at 4 °C, and then cultured for approximately 1 week in a growth chamber under an 8-h photoperiod (100 μmol m^−2^ s^−1^ light), ~70% relative humidity, and a temperature regime of 22 °C  ±  2 °C/18 °C  ±  2 °C. Thereafter, the seedlings were potted in soil.

The zygosity of the *mpc1* mutant was examined by PCR using the primer pairs *MPC1-LP/−RP*, *MPC1-LP*/LBb1.3, and *MPC1-RP*/LBb1.3 (sequences given in Additional file 1: Table S1). To generate *AtMPC1*-overexpressing (OE) lines, the *AtMPC1* coding sequence was amplified from cDNA of Col-0 using the primer pair *MPC1-OE-F/R* (Additional file 1: Table S1) and then inserted into the *pB2GW7.0* plasmid using Gateway™ recombination ligase (Invitrogen, Carlsbad, CA, USA). The construct was introduced into *Agrobacterium tumefaciens* strain GV3101 and transformed into Col-0 using the floral dip technique [28]. *MPC1/mpc1* complementation (C) lines were generated by replacing the *GUS* sequence in the plasmid *proMPC1::GUS* (described below) with the *AtMPC1* coding sequence amplified from Col-0 cDNA using the primer pair *MPC1-C-F/R* (Additional file 1: Table S1) to generate *proMPC1::MPC1.* The construct was introduced into the *mpc1* mutant by agroinfection as described above.

### Measurement of stomatal aperture

Stomatal aperture assay was performed as described [18]. After incubation of detached leaves from 4-week-old plants in closure buffer (1 mM CaCl_2_, 20 mM KCl, 5 mM MES-KOH, pH 6.15) for 2.5 h in light, various concentrations of either ABA (1, 10, and 50 μM) or pyruvate (10, 100, and 1000 μM) were added, and the leaves were incubated for a further 2.5 h. Ethanol and water were used as controls for the ABA and pyruvate treatments, respectively. Abaxial epidermal strips were then peeled and photographed under a light microscope. The stomatal pore widths and lengths were measured using ImageJ v. 1.37 (https://imagej.nih.gov/ij/), and the stomatal aperture was calculated as the ratio of the inner pore width/pore length of each pair of stomata [29–31]. More than 60 guard cells were calculated for each sample and all experiments were repeated for three times.

### Drought stress and measurement of water loss

For drought stress experiment, seeds were incubated in mixed soil (nutrient soil: vermiculite, 2: 1, *v*/v) in a growth chamber with sufficient watering. Approximately 3 weeks later, the plants were subjected to drought stress treatment by withholding water for 2–3 weeks. The plants were then rehydrated for 3 days. The effect of the stress was regularly monitored photographically. To quantify water loss, rosette leaves were detached from 4-week-old plants (three replicates per treatment and genotype), weighed, and placed on dry filter paper in light. The rosette leaves were weighed at a series of time points for 4 h at room temperature. The rate of water loss was calculated from the measured loss in fresh weight.

### GUS staining

Histochemical GUS staining was used to analyze the expression profile of *AtMPC1*. The native *AtMPC1* promoter was amplified using the primer pair *MPC1-GUS-F/R* (Additional file 1: Table S1) and inserted into the *pCAMBIA-ubiGUS* vector [32] to obtain the construct *proMPC1::GUS*, which was introduced into *Agrobacterium tumefaciens* strain GV3101 and from there into *A. thaliana* via the floral dip technique. Various organs of transgene homozygotes selected from the T_3_ generation were subjected to GUS staining by incubation at 37 °C for 4–6 h in 0.5 M phosphate buffer (pH 7.2) containing 100 mM K_4_Fe(CN)_6_, 100 mM K_3_Fe(CN)_6_, 10% *v*/v Triton X-100, 0.5 M EDTA, and 0.5% *w*/*v* X-Gluc. After staining, the material was bleached in 100% ethanol and monitored by light microscopy.

### Electrophysiology

*A. thaliana* guard cell protoplasts were isolated as described previously [33]. Briefly, the *A. thaliana* abaxial epidermis was peeled from 10 to 12 expanded young leaves of 4-week-old plants. The epidermis was homogenized in distilled water for 28 s and filtered through a 100-μm nylon mesh. The peels were then transferred into 2 mL enzyme solution I, which contained 0.7% Cellulysin cellulase, 0.1% PVP-40, and 0.25% BSA in 55% basic solution (5 mM MES, 0.5 mM CaCl_2_, 0.5 mM MgCl_2_, 0.5 mM ascorbic acid, 10 μM KH_2_PO_4_, 0.55 M sorbitol, pH 5.5). The peels were placed in a shaking water bath for 30 min to digest. Another 2 mL basic solution was added, and shaking was continued for a further 8 min. The peels were then filtered through a 100-μm nylon mesh and transferred into 2 mL enzyme solution II, which contained 1.5% Onozuka cellulase RS, 0.02% cellulase Y-23, and 0.25% BSA in 100% basic solution. The peels were shaken for at least 20 min to digest. After digestion, the peels were mixed by pipetting up and down with a 1-mL pipette and filtered through 30-μm nylon mesh. The protoplasts were obtained by centrifugation at 800 rpm for 5 min and washed twice with basic solution.

The whole-cell mode patch clamp experiment was performed as described previously [6, 34, 35]. To record the slow-type anion channel current, the bath solution contained 30 mM CsCl, 2 mM MgCl_2_, 1 mM CaCl_2_, and 10 mM MES (pH 5.6), and the pipette solution contained 150 mM CsCl, 2 mM MgCl_2_, 6.7 mM EGTA, 3.35 mM CaCl_2_, and 10 mM HEPES (pH 7.5). The osmolarities of the bath and pipette solutions were adjusted with sorbitol to 480 and 500 mOsm, respectively. Before using pipette solution, ATP (Mg-ATP, 10 μM) and GTP (10 μM) were added. The anion channel currents were recorded using the Axopath-200B amplifier (Molecular Devices, Downingtown, PA, USA) after the whole-cell configuration was achieved. The holding potential was +30 mV, and voltage steps were applied from −145 to +35 mV in +30 mV increments, with a duration of 60 s for every test voltage. To acquire and analyze the anion currents, pCLAMP software (version 10.2; Axon Instruments, Sunnyvale, CA, USA) was used, and SigmaPlot 11.0 (Systat Software, Richmond, CA, USA) was used to draw the current–voltage plots. For ABA or pyruvate treatment, guard cell protoplasts were exposed to 50 μM ABA or 100 μM pyruvate for 2 h before measurement. ABA/pyruvate was also added to both the bath and pipetting solutions.

### RT-PCR and quantitative real-time PCR (qPCR)

The transcript levels of *AtMPC1* and *NRGA1* were monitored by RT-PCR. Total RNA was isolated from Col-0, *mpc1* mutant, OE lines, C lines, *nrga1* mutant, and *mpc1/nrga1* double mutant using TRIzol reagent (Roche, Basel, Switzerland), and cDNA was synthesized using the ReverAid First Strand cDNA Synthesis Kit (Thermo Fisher, Waltham, MA, USA). qPCR amplifications were performed using the CFX96 Touch™ Real-Time PCR Detection system (Bio-Rad, Hercules, CA, USA, based on SYBR Premix Ex Taq mix (Roche)) with gene-specific primers for MPC1 and the internal control (ACTIN2). The primer sequences used are listed in Additional file 1: Table S1.

### Co-immunoprecipitation assay

The co-immunoprecipitation experiment was performed as described by Choi [36], with some modifications. Briefly, the ORF sequences of *AtMPC1* and *NRGA1* were amplified by PCR using primer pairs *MPC1*-Pro-F/R and *NRGA1*-Pro-F/R (sequences given in Additional file 1: Table S1) and cloned into the pCM1307-Flag and pCM1307-C-MYC vectors, respectively. Then, pCM1307-MPC1-Flag and pCM1307-NRGA1-MYC were transformed into *Agrobacterium* strain (*Agrobacterium tumefaciens* strain GV3101) and suspended to OD_600_  =  0.8 in infiltration buffer. Equal volumes of solutions of *Agrobacterium* carrying the two constructs, respectively, were mixed and co-infiltrated into the 3-week-old leaves of tobacco plants. The infiltrated tobacco plants were grown for an additional 3 days in a growth chamber at 28 °C. Proteins were then extracted from leaf samples weighing approximately 1 g using 2 mL extraction buffer (50 mM Tris-HCl, pH 8.0, 2 mM EDTA, 150 mM NaCl, 1 mM dithiothreitol, 10% glycerol, 1% Triton X-100, 1 mM PMSF, 1× protease inhibitor cocktail). The samples were placed on ice with gentle shaking for 1 h to solubilize membrane proteins and centrifuged at 13000  ×  *g* for 20 min. Supernatants were incubated with 10 mL anti-Flag polyclonal antibody (CWBio, Beijing, China) for 2 h at 4 °C with gentle rotation, and then 50 mL 50% (*v*/v) protein A agarose bead (Thermo Fisher Scientific, Waltham, MA, USA) slurry were added followed by incubation overnight. The beads were then washed four times with washing buffer (1 × PBS, 0.5% Triton X-100, 1 × protease inhibitor cocktail). After the last centrifugation, PBS was removed completely. The pellet was resuspended in 2× SDS-PAGE loading buffer. Eluted proteins were analyzed by immunoblotting using an anti-MYC antibody (CWBio, Beijing, China), followed by signal detection using the SuperSignal West Pico Chemiluminescent kit (Thermo Fisher Scientific, Waltham, MA, USA).

### Measurement of pyruvate content

*A. thaliana* ~15-day-old seedlings grown on solidified agar plates were incubated in 1/2 MS liquid medium for 24 h, after which either 50 μM ABA was added to the medium, or the seedlings were removed from the medium and placed on dry filter paper. After a 3-h exposure to one of these treatments, pyruvate was extracted and quantified following the method of Yu et al. with some modifications [37]. Briefly, 0.2 g sample (fresh weight) was ground in liquid nitrogen. The powder was mixed with 1 mL extraction solution (50% acetonitrile) and extracted overnight at 4 °C. The solution was then centrifuged at 13800  ×  *g* for 10 min. The supernatant was removed, and the remaining sample was extracted again in 0.5 mL extraction solution for 3 h at 4 °C. The two extracts were combined and analyzed using the L3000 HPLC device (Rigol, Beijing, China) equipped with a C18 chromatographic column. The mobile phase was 0.1% phosphate buffer (pH 2.3) at a flow rate 0.8 mL /min, a temperature of 35 °C, and an input volume of 20 μL.

### Fluorescence detection of ROS production

ROS levels in the guard cells were quantified using the fluorescent dye CM-H_2_DCFDA (Life Technologies, Carlsbad, CA, USA) as described previously [38, 39] with minor modifications. Briefly, the *A. thaliana* abaxial epidermis was peeled from expanded leaves of 4-week-old plants and incubated in buffer (1 mM CaCl_2_, 20 mM KCl, 5 mM MES-KOH, pH 6.15) for 2.5 h in light. Then, 50 μM ABA or 100 μM pyruvate was added to the buffer for a further 2.5 h. The epidermis was then incubated with 1‰ fluorescent dye CM-H_2_DCFDA for 20 min in the dark. Excess dye was removed by washing at least three times with distilled water. Fluorescence images were captured by confocal laser-scanning microscopy (LSM 700; Carl Zeiss, Oberkochen, Germany) at an excitation wavelength of 488 nm. The fluorescence intensities were measured using ImageJ v. 1.37 (https://imagej.nih.gov/ij/).

## Results

### Expression pattern of *AtMPC1* in *A. thaliana*

**Fig. 1:**
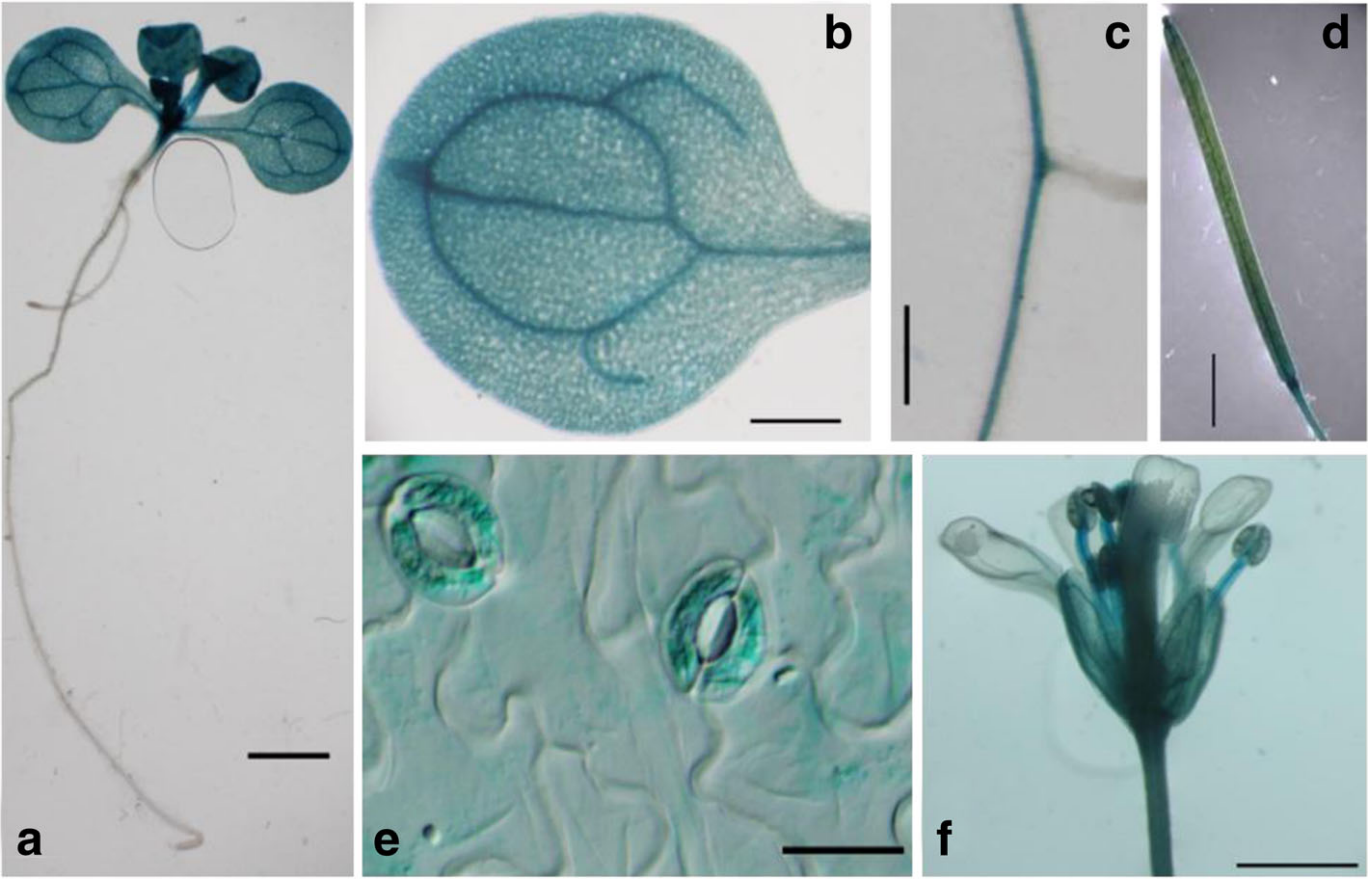
GUS staining in plants harboring *proAtMPC1::GUS.* **a** Two-week-old seedling. Bar: 1 mm. **b** Rosette leaf. Bar: 0.5 mm. **c** Root. Bar: 0.5 mm. **d** Silique. Bar: 3 mm. **e** Guard cells. Bar: 20 μm. **f** Inflorescence. Bar: 1 mm

Previously, the pyruvate carrier function of AtMPC1 in the yeast mutant *mpc1Δ* was confirmed by complementation analysis [18], and co-localization of GFP-tagged MPC1 and RFP-tagged NRGA1 indicated that AtMPC1 was deposited in the mitochondria, co-localizing with NRGA1 [27]. To further examine the tissue expression pattern of *AtMPC1* at the whole-plant level, transgenic plants harboring a GUS reporter gene fused to the *AtMPC1* native promoter were generated. GUS staining indicated that *AtMPC1* was ubiquitously expressed in the leaf, root, silique, and flowers, with a particularly high expression level in epidermal guard cells (Fig. 1), which suggests important functions of AtMPC1 in different tissues, especially in guard cells of the epidermis.

### AtMPC1 negatively regulated stomatal closure and the drought response

**Fig. 2:**
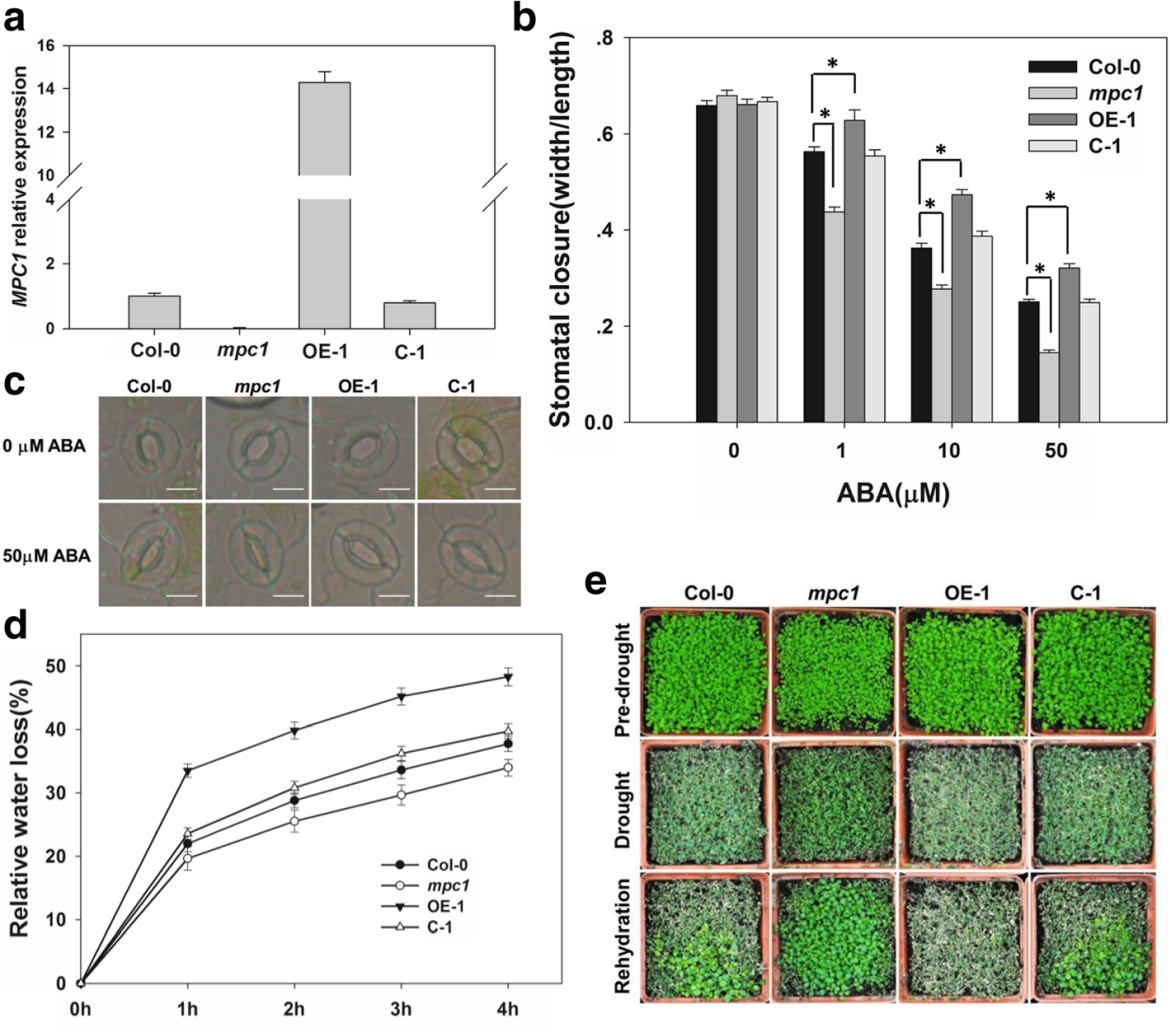
The involvement of AtMPC1 in stomatal movement and drought response. **a** The transcript level of *AtMPC1* was identified by qPCR in the *mpc1* mutant, the overexpressing line OE-1, and the complementation line C-1. **b** ABA-induced stomatal closure according to a width/length ratio analysis of stomatal aperture. Values are means ± SE (*n* = 3). Before width/length ratio analysis, at least 30 stomata were measured. *: significantly different at *P* < 0.05. **c** Representative stomata from plants untreated or exposed to 50 μM ABA. Bar: 10 μm. **d** The rate of water loss from the detached leaves of various transgenic lines. **e** The effect of withholding water from soil-grown Col-0, *mpc1*, OE-1, and C-1 plants

*AtMPC1* tissue expression was particularly abundant in guard cells, suggesting a potential role of AtMPC1 in the regulation of stomatal movements. To examine the physiological function of AtMPC1, we obtained the T-DNA insertion mutant *mpc1* (SALK_008945) (Fig. 2a, Additional file 1: Figure S1A, B and C). OE and C lines were also generated (Fig. 2a, Additional file 1: Figure S1D and S2A). The function of AtMPC1 was characterized by comparing the behavior of Col-0 with those of the *mpc1* mutant and the OE and C lines, focusing on the response to exogenous ABA. In plants exposed to a range of ABA concentrations (0, 1, 10, 50 μM), stomatal aperture was reduced in the mutant compared with Col-0 (Stomatal width/length ratio in Col-0: 0.658  ±  0.01, 0.563  ±  0.01, 0.362   ±   0.01, and 0.25  ±  0.005, respectively; In *mpc1*: 0.679  ±  0.011, 0.437  ±  0.01, 0.276  ±  0.008, and 0.145  ±  0.005, respectively), but stomatal closure in the OE lines was less sensitive to ABA (Stomatal width/length ratio in OE-1: 0.66  ±  0.011, 0.628  ±  0.022, 0.473  ±  0.011, and 0.321  ±  0.009, respectively). The level of ABA sensitivity in the C lines was similar to that in Col-0 (Stomatal width/length ratio in C-1: 0.666  ±  0.009, 0.554  ±  0.013, 0.387  ±  0.011, and 0.248  ±  0.007, respectively) (Fig. 2b and c, Additional file 1: Figure S2B, Additional file 2: Table S1). As the bulk of transpired water escapes via the stomata, its rate of loss from a detached leaf was lower in the mutant than in Col-0 (water loss rates in 4 h of 37.765%  ±  1.205 and 33.991%  ±  1.331 for Col-0 and *mpc1*, respectively), while the leaves of the OE line showed more rapid transpiration (water loss rate in 4 h of 48.27%  ±  1.408 for OE-1) (Fig. 2d, Additional file 1: Figure S2C, Additional file 2: Table S2). When water was withheld from soil-grown plants, *mpc1* appeared to be more tolerant to drought stress than did Col-0, while the OE plants remained withered even after re-watering (Fig. 2e, Additional file 1: Figure S2D and S3). The overall conclusion was that AtMPC1 plays a negative role in ABA-induced stomatal closure, thereby influencing the ability of the plant to tolerate drought stress.

### AtMPC1 was involved in the regulation of slow-type anion channels

**Fig. 3:**
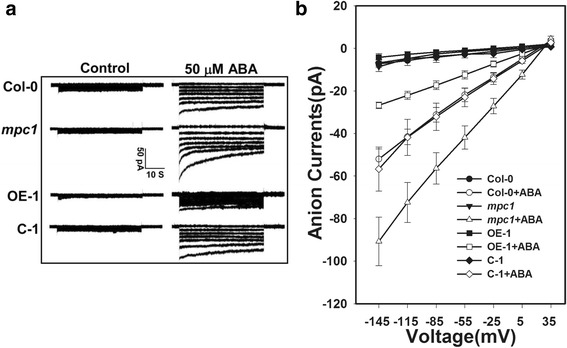
AtMPC1 affected the anion channel activity in guard cells. **a** Patch clamp whole-cell recordings of the anion currents in Col-0, *mpc1*, OE-1, and C-1 protoplast guard cells in the presence/absence of 50 mM ABA. **b** Current/voltage relationships of whole-cell slow-type anion currents, as illustrated in (**a**). The numbers of guard cells measured were as follows: Col-0, *n* = 9; Col-0+ABA, *n* = 9; *mpc1*, *n* = 10; *mpc1*+ABA, *n* = 13; OE-1, *n* = 6; OE-1+ABA, *n* = 13; C-1, *n* = 9; and C-1+ABA, *n* = 9. Values are means ± SE

As ABA activates slow-type anion currents in guard cells [40], it was of interest to determine the effects of altering ABA sensitivity via manipulation of *AtMPC1* expression on the performance of slow-type anion channels. In response to ABA treatment, the size of the anion current in the guard cells increased more substantially in the *mpc1* mutant than in Col-0. The guard cell anion current in the C lines behaved similarly to that in Col-0, but in the OE lines, the currents were markedly lower than those in Col-0 (Fig. 3, Additional file 1: Figure S4, Additional file 2: Table S4). These responses confirmed that AtMPC1 acts as a negative regulator of ABA-enhanced slow anion channel function during stomatal closure.

### AtMPC1 interacted with NRGA1

**Fig. 4:**
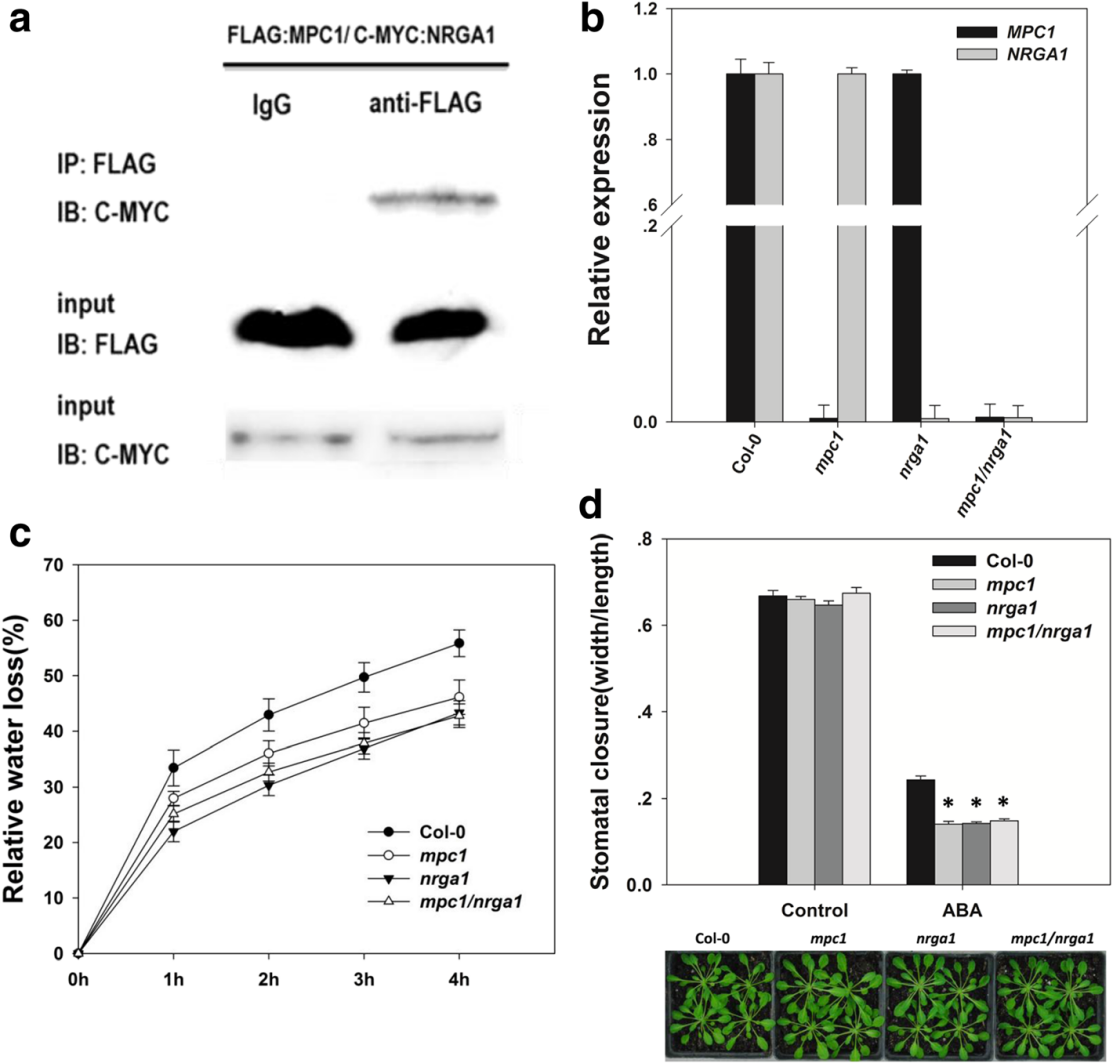
AtMPC1 and NRGA1 formed a heterocomplex. **a** In vivo co-immunoprecipitation assay revealed an interaction between AtMPC1 and NRGA1 in *N. benthamiana* leaves. IP: Immunoprecipitation; IB: Immunoblotting. **b** Identification of *mpc1/nrga1* based on qPCR. **c** The rate of water loss from detached leaves of the double mutant. **d** Stomatal movement in the double mutant in response to treatment with 50 μM ABA and images of the rosettes of Col-0, *mpc1*, *nrga1*, and *mpc1/nrga1* at the age used for the stomatal aperture experiments. Values are means ± SE (*n* = 3). Before width/length ratio analysis, at least 30 stomata were measured

The physiological experiments indicated that AtMPC1 has a function similar to that of NRGA1 in the regulation of stomatal closure, and co-expression of *NRGA1* with *AtMPC1* restored the growth of the yeast mutant *mpc2Δ/mpc3Δ* [18]. Therefore, it is necessary to explore the relationship between AtMPC1 and NRGA1 by molecular and genetic methods. Co-immunoprecipitation using an anti-Flag antibody demonstrated an interaction between AtMPC1 and NRGA1 in plants (Fig. 4a). We also obtained the double mutant *mpc1/nrga1* by hybridization of *mpc1* with *nrga1* (Fig. 4b), and this mutant did not display an extreme phenotype compared with those of the *mpc1* and *nrga1* single mutants, regardless of stomatal movement (Stomatal width/length ratio: 0.243  ±  0.009, 0.141  ±  0.006, 0.142  ±  0.004, and 0.148  ±  0.005 for Col-0, *mpc1*, *nrga1*, and *mpc1/nrga1*, respectively, after 50 μM ABA treatment) or the rate of water loss (water loss rates in 4 h of 55.83%  ±  2.397, 46.116%  ±  3.098, 43.312%  ±  2.183, and 42.783%  ±  2.124 for Col-0, *mpc1*, *nrga1*, and *mpc1/nrga1*, respectively) (Fig. 4c and d, Additional file 2: Table S1-S2). The assay of physiological function also indicated that AtMPC1 and NRGA1 form an interactional heterocomplex, consistent with previous reports in yeast and mammals [20, 41].

### ABA and drought stress induced pyruvate accumulation

**Fig. 5:**
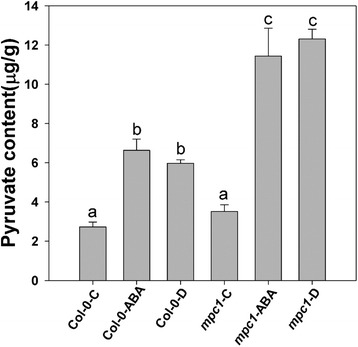
Response of tissue pyruvate content to ABA treatment or moisture stress in Col-0 and the *mpc1* mutant. Statistically significant (*P* < 0.05) differences are indicated by different letters. C: Control treatment; D: Moisture stress treatment

As a MPC, the activity of AtMPC1 is expected to influence the transport of pyruvate. Therefore, pyruvate contents were compared between Col-0 and the *mpc1* mutant under normal and stressed growth conditions (Fig. 5). When treated with ABA or exposed to drought stress, the pyruvate contents were increased in both Col-0 and *mpc1*. However, the mutant contained more pyruvate than did Col-0 under each of the treatment conditions (Fig. 5). Thus, both ABA and drought stress induced the accumulation of pyruvate in an AtMPC1-dependent manner.

### Pyruvate acted on stomatal closure by modulating slow-type anion currents

**Fig. 6:**
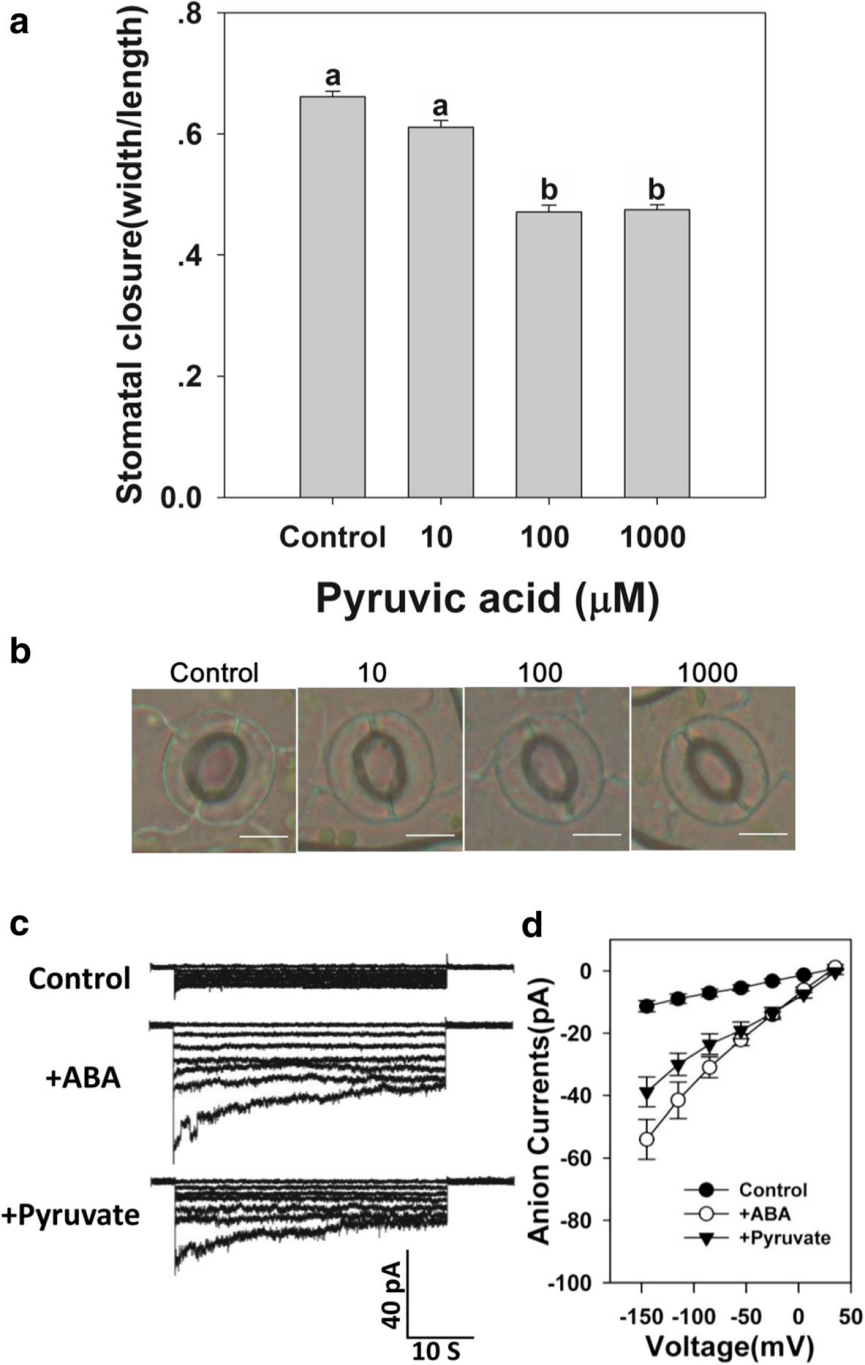
Pyruvate promoted stomatal closure by activating slow-type anion channel activity. **a** The induction of stomatal closure by 10–1000 μM pyruvate. Values are shown as means ± SE (*n* = 3). Before width/length ratio analysis, at least 30 stomata were measured. Statistically significant (*P* < 0.05) differences are indicated by different letters. **b** Representative stomata in control and pyruvate-treated plants. Bar: 10 μm. **c** Patch clamp whole-cell recordings of slow-type anion currents in guard cell protoplasts treated with either 50 μM ABA or 100 μM pyruvate. **d** Current/voltage relationships of whole-cell slow-type anion currents, as illustrated in (**c**). The numbers of guard cells measured were as follows: Control, *n* = 15; ABA, *n* = 11; and Pyruvate, *n* = 14. Values are means ± SE

As AtMPC1 is likely involved in ABA-induced stomatal closure, and ABA induces the accumulation of pyruvate, it was suggested that pyruvate may act as a regulator of stomatal movement. This is supported by the observation that pyruvate induced stomatal closure: the stomatal aperture decreased in size under higher concentrations of pyruvate until reaching 100 μM (Stomatal width/length ratio: 0.662  ±  0.009, 0.611  ±  0.011, 0.471  ±  0.011, and 0.474  ±  0.008 for Control, 10, 100, and 1000 μM pyruvate, respectively) (Fig. 6a and b, Additional file 2: Table S1). Exposure to either 50 μM ABA or 100 μM pyruvate increased the size of the anion current, although pyruvate was less effective than ABA (Fig. 6c and d, Additional file 2: Table S4). Besides, we also did patch clamp whole-cell recordings of slow-type anion currents in Col-0 guard cell protoplasts under control condition and treated both with 50 μM ABA and 100 μM pyruvate, to some extent, the currents were slightly increased compared to those of ABA or pyruvate treatment alone (Fig. 6c and d, Additional file 1: Figure S5, Additional file 2: Table S4). However, we still could not confirm if the elevation of cytosolic pyruvate could strengthen the ABA activated slow-type anion currents i.e., whether there was an additive effect in anion channel regulation. Therefore, additional research needs to be done in future.

### Pyruvate induced production of ROS

**Fig. 7:**
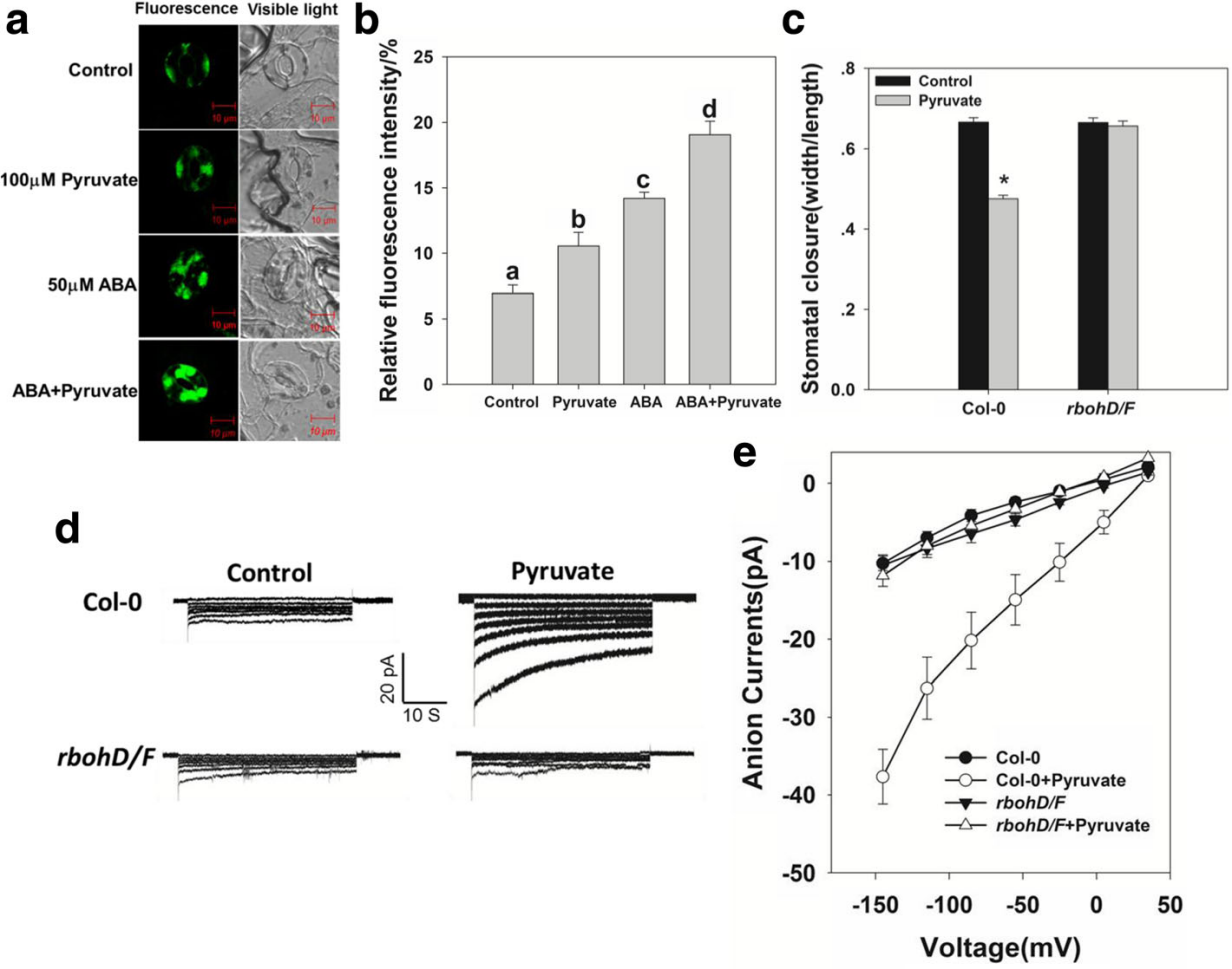
Pyruvate enhanced ROS production. **a** Exogenous ABA alone or pretreatment with ABA and exogenous pyruvate induced ROS production (labeled with CM-H2DCFDA) in guard cells. Bar: 10 μm. **b** The intensity of the fluorescence signal was measured using ImageJ. Values are means ± SE (*n* = 3). At least 30 guard cells were measured within each replicate. Statistically significant (*P* < 0.05) differences are indicated by different letters. **c** Pyruvate promoted stomatal closure in Col-0, but not the *rbohD/F* mutant. Values are means ± SE (*n* = 3). Before width/length ratio analysis, at least 30 stomata were measured. **d** Patch clamp whole-cell recordings of the anion currents in Col-0, *rbohD/F* protoplast guard cells in the presence/absence of 100 μM pyruvate. **e** Current/voltage relationships of whole-cell slow-type anion currents, as illustrated in (**d**). The numbers of guard cells measured were as follows: Col-0, *n* = 8; Col-0+Pyruvate, *n* = 10; *rbohD/F*, *n* = 7; *rbohD/F*+Pyruvate, *n* = 7. Values are means ± SE

ROS participate in the regulation of stomatal movement [42, 43], and mitochondrial metabolism influences the production of ROS. Therefore, we investigated the relationships between ROS and pyruvate-mediated stomatal movement and anion channel activity. As visualized by CM-H_2_DCFDA staining, ROS production was induced in guard cells exposed to either ABA or pyruvate. After ABA pretreatment, the fluorescence intensity was stronger than that after treatment with ABA or pyruvate alone, indicating that pyruvate induced the production of ROS (Fig. 7a and b, Additional file 2: Table S3). In addition, pyruvate induction of stomatal closure was disrupted in the double mutant *rbohD/F*, which failed to show ROS production (Stomatal width/length ratio without vs. with pyruvate treatment: 0.667  ±  0.011 and 0.475 ±  0.009 for Col-0, respectively, and 0.665  ±  0.012 and 0.657  ±  0.012 for *rbohD/F*, respectively) (Fig. 7c, Additional file 2: Table S1). Consistent with stomatal movement, pyruvate did not increase the size of the anion current in *rbohD/F* (Fig. 7d and e, Additional file 2: Table S4). These observations suggested that increased pyruvate-induced stomatal closure was dependent on ROS accumulation in guard cells.

## Discussion

MPCs are required to transport pyruvate into the mitochondria where it enters into the TCA cycle. Although transport of pyruvate into the mitochondrial has long been known to require a carrier-mediated process, it has taken several decades since to determine the molecular identity of the pyruvate carrier responsible for its mitochondrial localization [19, 20]. A great deal of research effort has been focused on the relationship between MPC activity and cancer because of the special metabolic phenomenon of the Warburg effect in cancer cells [24, 41, 44]. There have also been studies regarding the MPC activity and MPC complexes in yeast and mammals, the genomes of which have been shown to encode at least three and two MPCs, respectively [22, 45, 46]. Furthermore, the roles of MPCs in the modulation of respiratory capacity and stress tolerance have been reported in yeast [23], suggesting that MPC has multiple functions in both metabolism and stress responses.

In plants, the putative MPC2-like protein NRGA1 has been reported to be a negative regulator of guard cell ABA signaling and the drought response [18]. Another member of the MPC family, AtMPC1, has been confirmed to show pyruvate carrier activity in yeast and mitochondrial localization in plant cell [18, 27]. Here, we further explored the function of AtMPC1 through physiology, molecular biology, and genetic methods. Similar to *NRGA1*, *AtMPC1* was shown to be ubiquitously expressed in different plant tissues (Fig. 1), with especially high levels in guard cells (Fig. 1e). The negative roles of AtMPC1 in guard cell ABA signaling and the plant drought response are shown in Figs. 2 and 3. Due to the similar functions of NRGA1 and AtMPC1, we considered that they interact in plants (Fig. 4a), consistent with a previous report that the MPC family is composed of different members [19, 20]. The similar phenotypes between the double mutant *mpc1/nrga1* and either single mutant strengthened this hypothesis (Fig. 4b, c and d).

As a member of the MPC family, the absence of AtMPC1 would be expected to disturb pyruvate metabolism in plants. It has been reported previously that the capacity to alter the cytosolic level of pyruvate is a key determinant of stress tolerance [23]. Measurement of the tissue content of pyruvate showed that there were no differences between Col-0 and the *mpc1* mutant under control conditions (Fig. 5), which may have been due to the functional redundancy of other MPC members to maintain basic pyruvate metabolism. However, ABA and drought stress induced accumulation of pyruvate in both Col-0 and the *mpc1* mutant, and the pyruvate level was higher in the latter than in the former (Fig. 5). It was concluded that the loss of AtMPC1 impeded pyruvate metabolism in plants, especially under conditions of stress. Interestingly, administration of exogenous pyruvate induced stomatal closure in a concentration-dependent manner (Fig. 6a and b), and this process relied on the anion channel activity of guard cells (Fig. 6c and d). The reversibility of ABA-inhibited stomatal opening by pyruvate in the presence of ATP has also been reported previously [47], indicating the equalizing regulation of stomatal aperture by pyruvate to maintain balance between the demands of photosynthesis and transpiration under drought conditions; the mechanism of pyruvate induction of stomatal closure needs to be explored.

The transport of pyruvate into mitochondria is a vital step in energy metabolism, and the mitochondria represent a major source of ROS [48–50]. Certain ROS function as signaling molecules, particularly in the context of the abiotic stress response, and have been implicated in ABA-induced stomatal closure [51–55]. Here, ROS concentrations were increased under conditions of pyruvate treatment (Fig. 7a and b), and the induction of stomatal closure and activation of slow-type anion currents by pyruvate were impaired in the double mutant *rbohD/F* (Fig. 7c, d and e), which failed to show ROS production. Pyruvate was suggested to regulate stomatal closure indirectly by inducing ROS production. In addition to its role in oxidative metabolism, pyruvate is a branching point for the syntheses of glucose, lactate, fatty acids, and amino acids. Further studies are required to examine the roles of MPCs in plant sucrose and organic acid metabolisms, which have also been implicated in guard cell ABA signaling [56–58].

## Conclusion

**Fig. 8:**
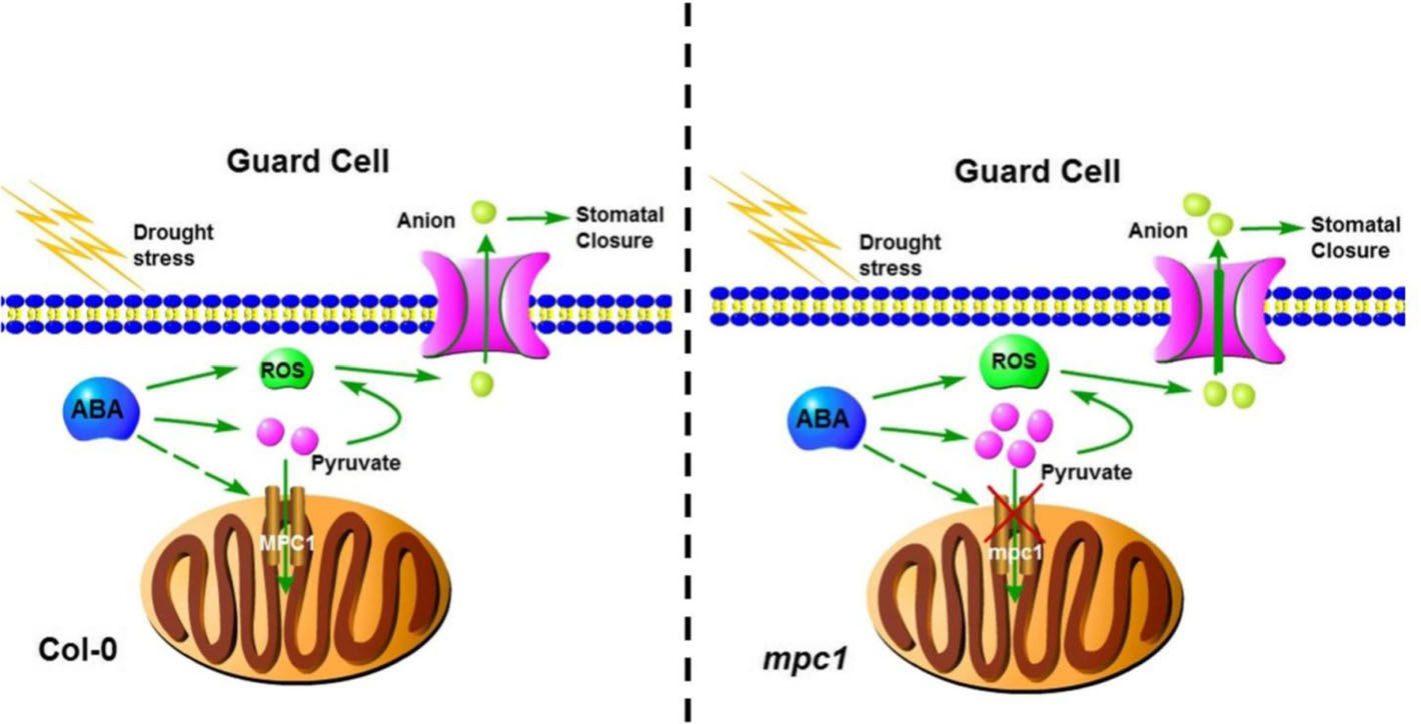
Proposed model for the role of AtMPC1 in the regulation of stomatal movement and the drought response

A working model for the role of AtMPC1 in the regulation of stomatal movement and the drought response is presented in Fig. 8. Plants exposed to drought stress responded by elevation of ABA, and the consequent increase in the ABA level induced elevation of the cellular pyruvate content, which in turn enhanced ROS accumulation to activate slow-type anion channels, finally inducing stomatal closure. AtMPC1 appears to reduce the pyruvate concentration by transporting it to mitochondria. In the *mpc1* mutant, pyruvate continued to accumulate in the cytosol, thereby encouraging the production of ROS and accelerating stomatal closure.

## Additional files

Additional file 1: Figure S1. Validation of the *mpc1* mutant, the overexpressing line OE-1, and the complementation line C-1; **Figure S2.** The phenotype of the overexpressing line OE-2 and complementation line C-2; **Figure S3.** Phenotypic analysis of Col-0 and the *mpc1* mutant under drought stress conditions; **Figure S4.** Slow-type anion channel activity in the overexpressing line OE-2 and complementation line C-2 guard cells; **Figure S5.** Patch clamp whole-cell recordings of slow-type anion currents in Col-0 guard cell protoplasts under control condition and treated with 50 μM ABA and 100 μM pyruvate; **Table S1.** List of primers used in this study. (ZIP 11739 kb)

Additional file 2: Table S1. Stomatal movement raw data; **Table S2.** Water loss rate raw data; **Table S3.** ROS fluorescence intensity raw data; **Table S4.** Patch clamp raw data. (ZIP 60 kb)

## Abbreviations

35S: Cauliflower mosaic virus 35S promoter
ABA: Abscisic acid
AtMPC1: *Arabidopsis thaliana* mitochondrial pyruvate carrier 1
C: Complementation
Col-0: Columbia-0
NRGA1: Negative regulator of guard cell ABA signaling 1
OE: Over-expression
ROS: Reactive oxygen species
TCA: Tricarboxylic acid cycle
